# Neoadjuvant Immunotherapy and De-escalation of Surgery in Locally Advanced Breast Implant-associated Anaplastic Large Cell Lymphoma

**DOI:** 10.1055/a-2427-2066

**Published:** 2024-12-24

**Authors:** Marzia Salgarello, Jaroslaw Krupa, Rebecca Allchin, Simon Pilgrim, Fiona Miall, Arianna Di Napoli, Maurizio Martelli, Giulio Tarantino

**Affiliations:** 1 Department of Plastic Surgery, Università Cattolica del Sacro Cuore, Rome, Italy; 2 Department of Breast Surgery, University Hospitals of Leicester NHS Trust, Leicester, Leicester, United Kingdom of Great Britain and Northern Ireland; 3 Department of Haematology, University Hospitals of Leicester NHS Trust, Leicester, United Kingdom of Great Britain and Northern Ireland; 4 Department of Clinical and Molecular Medicine, Sapienza University, Sant'Andrea University Hospital, Rome, Italy; 5 Group of Experts on BIA-ALCL, Istituto Superiore di Sanità, Roma, Lazio, Italy; 6 Department of Translational and Precision Medicine, Umberto I Policlinico di Roma, Roma, Lazio, Italy; 7 Department of Plastic, Reconstructive, and Esthetic surgery, Università Cattolica del Sacro Cuore, Rome, Italy

**Keywords:** brentuximab vedotin, breast implant-associated anaplastic large cell lymphoma, breast implant, neoadjuvant therapy, capsulectomy

## Abstract

Breast implant-associated anaplastic large cell lymphoma (BIA-ALCL) is a rare form of non-Hodgkin T-cell lymphoma diagnosed in patients with a history of breast implants. Most patients develop a periprosthetic effusion at early stages of disease while less common presentations include a palpable mass, severe capsular contracture, lymphadenopathy, or cutaneous erythema. Due to the complex nature of this disease, a multidisciplinary approach is necessary for optimal management, particularly in locally advanced disease or inoperable patients. We present the successful use of neoadjuvant therapeutic protocols in two cases of locally advanced BIA-ALCL. The first case was a 52-year-old patient with a left breast mass-like stage III disease who underwent combined targeted immunotherapy and chemotherapy (brentuximab vedotin [BV]–cyclophosphamide, doxorubicin, prednisone [CHP]). Following a complete radiological and metabolic response, the patient underwent bilateral implant removal, right total intact capsulectomy, left en bloc capsulectomy, and skin resection from the left inframammary fold in continuity with the capsule. The second case was a 65-year-old patient with right breast swelling and mass-like stage IIA disease who received targeted immunotherapy, BV. Following a complete metabolic response, she underwent bilateral implant removal and en bloc capsulectomy. A literature review and the reported cases suggest the effectiveness of targeted immunotherapy as monotherapy or in combination with chemotherapy in locally advanced BIA-ALCL in disease downstaging, surgical de-escalation, reduction of significant postoperative complications, and an acceptable tolerance profile. Although surgery is an essential part of treatment, the timing and type of intervention should be carefully planned, especially when primary, radical resection is uncertain.

## Introduction

Anaplastic large cell lymphomas (ALCLs) are a group of mature CD30+ T-cell lymphomas characterized by the proliferation of large and pleomorphic cells with similar immunophenotypic features but a variety of clinical characteristics. 1 ALCLs are divided into four distinct subtypes: anaplastic lymphoma kinase (ALK)-positive, ALK-negative, primary cutaneous, and breast implant-associated (BIA-ALCL). 1 BIA-ALCL is an uncommon CD30+ and ALK lymphoma typically occurring in women 8 to 10 years following breast implantation for breast augmentation or reconstruction. 2 In 1997, Keech and Creech reported the first case of ALCL in proximity to a textured saline-filled breast implant 6 years following bilateral breast augmentation. 3 As of January 2011, the U.S. Food and Drug Administration (FDA) declared a possible correlation between breast implants and ALCL, and in May 2016, the World Health Organization (WHO) recognized BIA-ALCL first as a provisional, and then in 2022 as a definitive T-cell lymphoma entity. 1 The current lifetime risk of BIA-ALCL, according to the American Society of Plastic Surgeons (ASPS), is estimated to be 1:2,207 to 1:86,029 for patients with textured implants. 4 Although the molecular pathogenesis is poorly established, numerous hypotheses have been suggested including genetic predisposition, subclinical bacterial infection, and a chronic inflammatory response following long-term immune stimulation to breast implants. 5 The most common clinical presentation is a late onset of a periprosthetic effusion and may be associated with breast distortion, swelling, and asymmetry. Less common presentations may be a palpable mass, severe capsular contracture, lymphadenopathy, or cutaneous erythema. 6 Due to the complex nature of this disease, a multidisciplinary approach involving medical oncologists, hematopathologists, surgical oncologists, and plastic surgeons is recommended for the management of patients with BIA-ALCL, particularly in case of advanced disease or inoperable patients, with the aim of providing the best diagnostic work-up, treatment, and surveillance strategies. The current National Comprehensive Cancer Network (NCCN) consensus guidelines indicate bilateral breast implant removal with en bloc surgical resection of the surrounding capsule in patients with disease limited to the capsule. 7 According to FDA regulations, the prophylactic explantation of textured implants is not recommended in asymptomatic BIA-ALCL patients; nevertheless, patients may discuss the benefits and drawbacks of implant removal with their health care providers to make an informed decision about their health. 8 9

The use of adjuvant chemotherapy or radiation therapy is considered for patients with local residual disease, positive margins, or surgically unresectable disease. Systemic therapies include brentuximab vedotin (BV), an anti-CD30 monoclonal antibody, anthracycline-based chemotherapeutic regimen CHOP (cyclophosphamide, adriamycin, vincristine, and prednisone) or CHOEP (cyclophosphamide, adriamycin, vincristine, etoposide, and prednisone), or a combination of both are reserved for cases of residual or disseminated disease (stage II–IV). There may, however, be a role for neoadjuvant therapy in advanced cases of BIA-ALCL. Italian Ministry of Health guidelines on the diagnosis and treatment of BIA-ALCL, recently published in November 2022, indicate the use of systemic chemotherapy, BV, or a combination of both in the neoadjuvant setting in cases of stage IV disease. 10 Until now, few cases in the literature have reported the use of neoadjuvant therapy for the treatment of locally advanced or disseminated diseases. We present two clinical cases reporting the successful use of neoadjuvant therapeutic protocols in locally advanced BIA-ALCL. The first case reports a 52-year-old patient with a left breast mass-like stage III disease who underwent combined targeted immunotherapy and chemotherapy (BV-cyclophosphamide, doxorubicin, prednisone [CHP]). Following a complete radiological and metabolic response, the patient underwent bilateral implant removal, right total intact capsulectomy, left en bloc capsulectomy, and skin resection from the left inframammary fold in continuity with the capsule. The second case reports a 65-year-old patient with right breast swelling and mass-like stage IIA disease who received targeted immunotherapy with BV. Following a complete metabolic response and a significant reduction in mass size, she underwent bilateral implant removal and en bloc capsulectomy. Both patients provided written informed consent for the publication and use of their images.

## Case

### Case 1

**Fig. 1:**
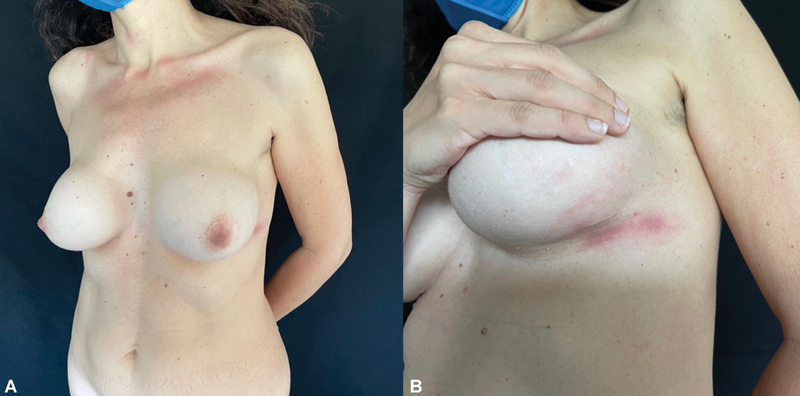
Preoperative clinical photographs in a 50-year-old patient with BIA-ALCL of the left breast ( **A** ). Our patient in June 2022 prior to neoadjuvant therapies and surgical treatment. ( **B** ) A close-up view of the cutaneous erythema of the lateral portion of the left inframammary fold. BIA-ALCL, breast implant-associated anaplastic large cell lymphoma.

**Fig. 2:**
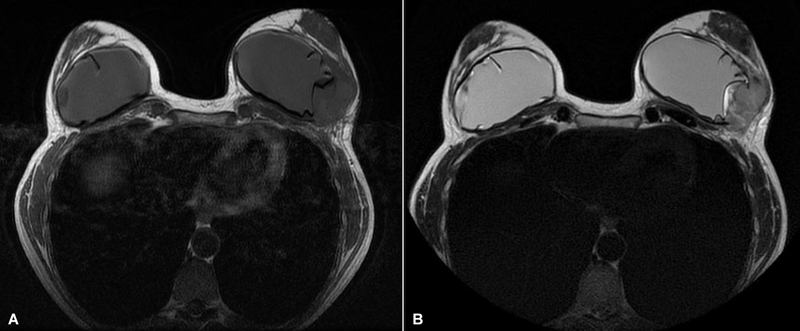
Axial images from a breast MRI. ( **A** ) Contrast-enhanced T1-weighted image shows a mass-like enhancement located on the lateral aspect of the left breast implant. ( **B** ) Contrast-enhanced T2-weighted image shows the same mass-like enhancement.

**Fig. 3:**
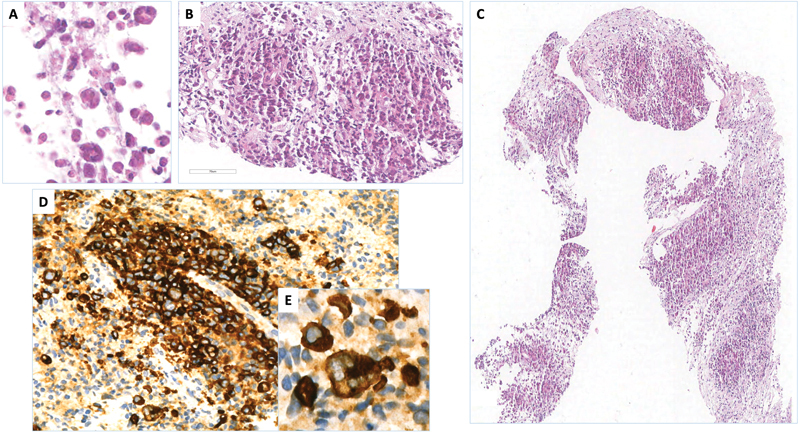
Histology of the needle biopsy of the pericapsular mass showing aggregates of large atypical CD30+ cells. ( **A–C** ) Haematoxylin and eosin, original magnification (o.m.) ×400, ×300, and ×72. ( **D, E** ) Immunostaining for CD30, o.m. ×350 and ×1,000.

**Fig. 4:**
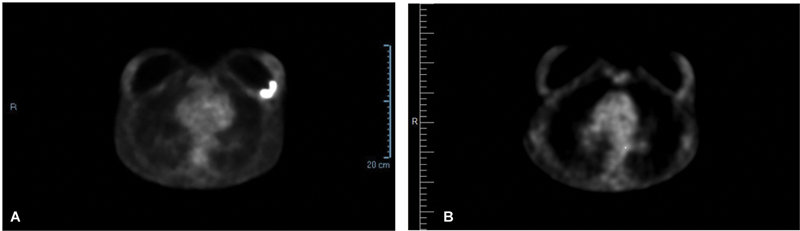
PET-CT scan images taken before and after neoadjuvant chemotherapy and targeted therapy. ( **A** ) Total body PET-CT scan taken in June 2022 showed a metabolically active mass on the posterolateral aspect of the left breast. ( **B** ) Total body PET-CT scan taken in November 2022 showed a complete metabolic response.

**Fig. 5:**
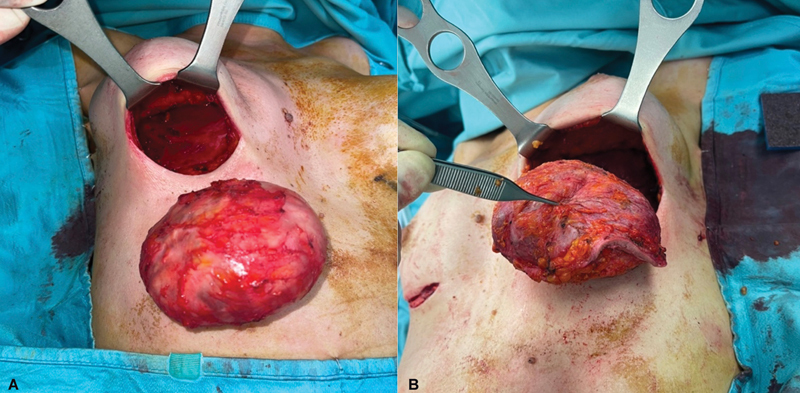
The patient underwent surgery to have both breast implants and capsules removed. ( **A** ) Right total intact capsulectomy. ( **B** ) Left en bloc capsulectomy together with skin resection from the left inframammary fold in continuity with the capsule.

**Fig. 6:**
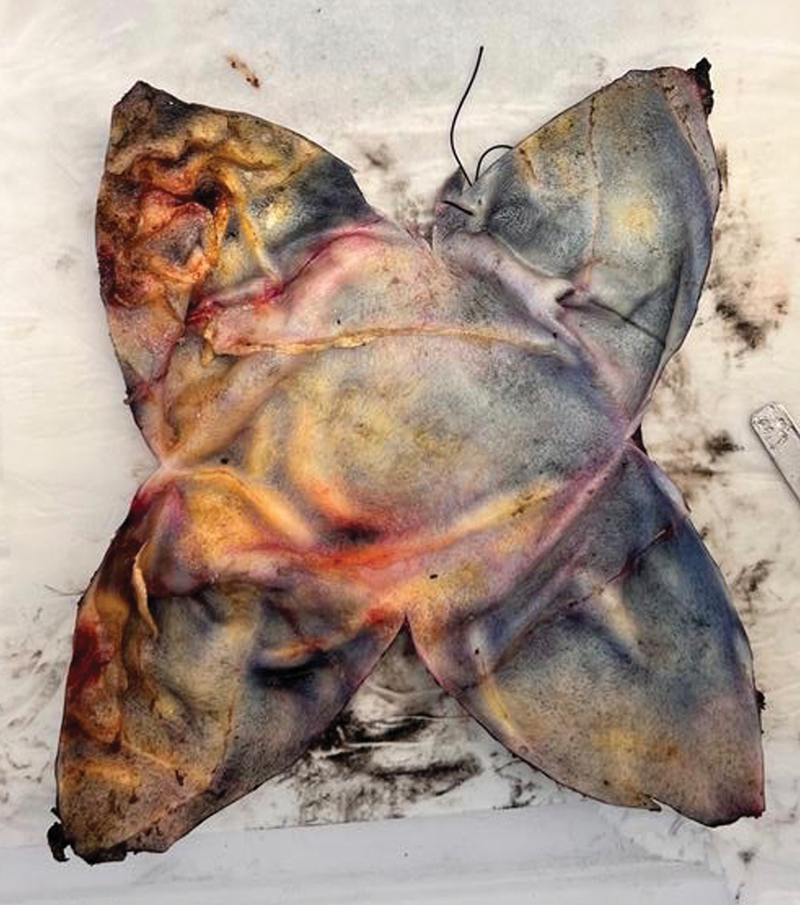
Pathological examination of the left breast capsule.

**Fig. 7:**
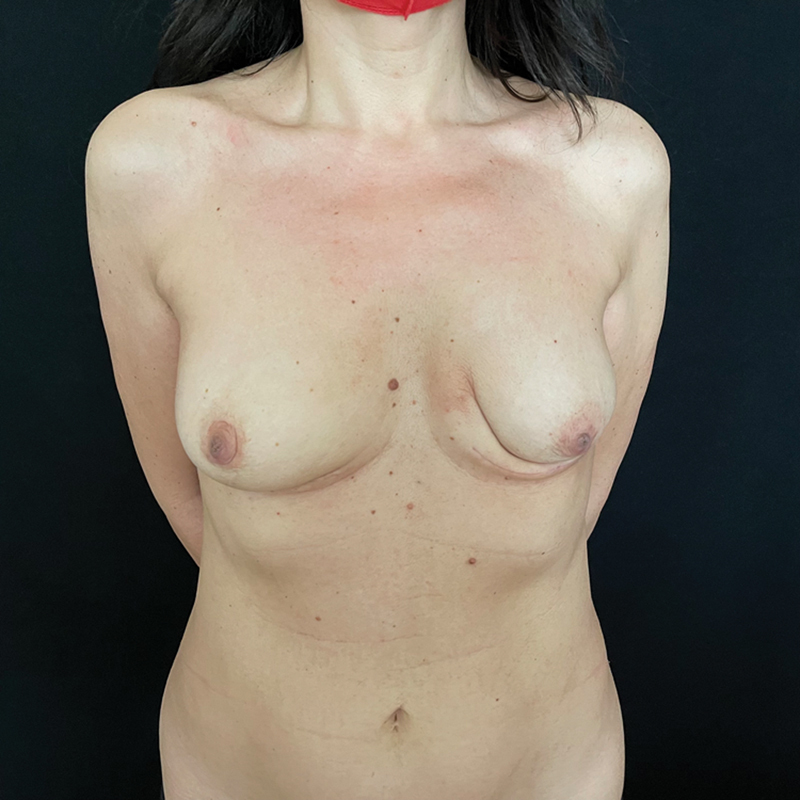
Clinical photograph taken 3 months following surgery.

A 52-year-old woman underwent bilateral subglandular breast augmentation in 1998 with macrotextured silicone breast implants (Silimed 220 mL). She did not undergo breast implant replacement since primary implantation. Physical examination showed bilateral inframammary fold scars consistent with her previous breast surgery. The left breast was slightly larger than the contralateral and a palpable breast mass in the lower-outer quadrant was felt fixed to the implant capsule. Previous surgical procedures included rhinoseptoplasty in 1989 and the removal of a fibroadenoma of the left breast in 2006. Her past medical history included multiple thyroid nodules, sideropenic anemia, and hiatal hernia. Daily pharmacological therapy included pantoprazole, levothyroxine, sulfamethoxazole, and trimethoprim for *Pneumocystis jirovecii* pneumonia prophylaxis during the 4 months of chemotherapy administration. Our patient developed left breast heaviness and mastalgia as of December 2021. Breast and axillary cavity ultrasound as well as 3D mammography failed to document pathological findings. By February 2022, the patient noticed a palpable breast mass in the lower-outer quadrant of the left breast followed by cutaneous erythema of the lateral aspect of the left inframammary fold in March 2022 ( Fig. 1 ). She underwent a breast MRI which did not show significant pathological findings. By June 2022, after the failure of clinical improvement following an antibiotic and anti-inflammatory treatment, a second breast MRI noted multiple radial breast implant folds bilaterally and an area of altered mass-type enhancement approximately 5.0 cm × 4.0 cm × 4.2 cm located in the lower-outer quadrant of the left breast and appeared fixed to the lateral aspect of the left breast implant ( Fig. 2 ). No focal or diffuse areas with altered enhancement were noted in the right breast parenchyma. Multiple abnormal lymph nodes were observed in the left axilla, the largest of which measured 1.8 cm × 1.2 cm, while no lymph nodal abnormalities were present in the right axilla. Ultrasound-guided Tru-cut needle biopsy of the left periprosthetic mass documented morphologic and immunohistochemical features (CD30 + , ALK-, CD3-, CD5-, PAX5-, LMP1, cytokeratin AE1/AE3-, S100-) compatible with a BIA-ALCL ( Fig. 3 ). Core biopsy from the left axillary lymph node documented fragments of a lymph node structure with rare CD30+ large cells suspicious but not conclusive for lymph node involvement of BIA-ALCL. One week later, a whole body PET-CT scan documented a 5 × 4 × 2 cm metabolically active mass on the posterolateral aspect of the left breast, multiple active lymph nodes in the ipsilateral axilla, left internal mammary lymph node chain and left subpectoral lymph nodes (stage III, T4N2M0). Given the extent of surgery necessary to achieve complete excision of pathological tissue, neoadjuvant chemotherapy combined with targeted therapy was administered. From July to October 2022, she underwent a total of six cycles of neoadjuvant chemotherapy (doxorubicin, cyclophosphamide, and prednisone) of which the second, third, and fourth cycles were combined with BV. Immunotherapy was interrupted following the fourth cycle due to peripheral neuropathy. Following the completion of four cycles of chemotherapy in September 2022, the total body CT scan no longer documented pathological enhancement of the left breast periprosthetic mass as well as a significant reduction in size of the pathological axillary, subpectoral, and internal mammary chain lymph nodes. After completion of six cycles of neoadjuvant chemotherapy, total body PET-CT scan in November 2022 showed a complete metabolic response, and on December 1, 2022, breast MRI demonstrated a total T response and a partial N response ( Fig. 4 ). On December 12, 2022, the patient underwent bilateral implant removal, right total intact capsulectomy, left en bloc capsulectomy with a 6 cm × 0.7 cm cutaneous resection from the left inframammary fold corresponding to the site of the previous erythema ( Fig. 5 ). Initial biopsy of axillary lymph node showed suspicious features, but not conclusive for lymph node involvement. Given the excellent response to neoadjuvant therapy (complete metabolic response on PET-CT), it was deemed acceptable to avoid the surgery to the axilla and the risk of lymphedema. Close follow-up and interval ultrasound scans were offered, and alternative options were discussed with the patient. Pathological examination and immunohistochemistry of the breast capsule and skin resection showed no signs of residual disease ( Fig. 6 ). In view of an excellent preoperative radiological response and absence of residual disease in the surgical specimen, no adjuvant radiotherapy or targeted therapy was recommended. Thus far, she has had a complete response and an unremarkable postoperative recovery without complications ( Fig. 7 ). The patient has shown no evidence of disease reoccurrence after 12 months following surgery.

### Case 2

**Fig. 8:**
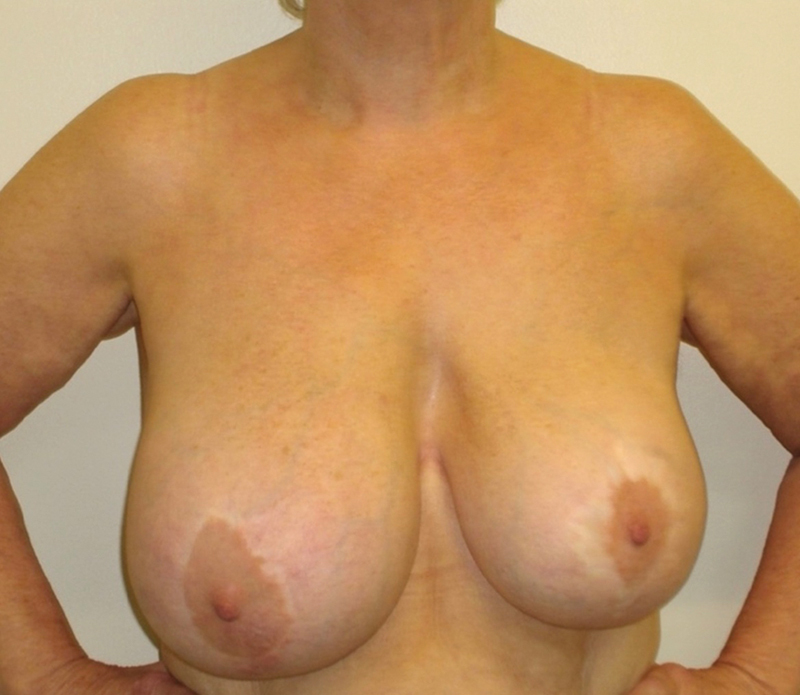
Clinical photographs at presentation. Visible breast asymmetry, large seroma, and palpable mass in the right upper-inner quadrant.

**Fig. 9:**
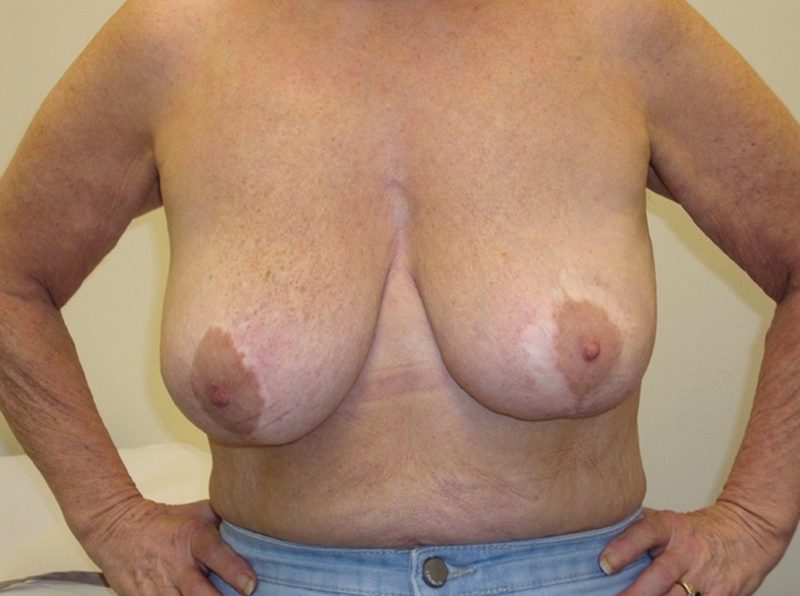
Clinical photograph on the 19th postoperative day (previously included in an early report).

**Fig. 10:**
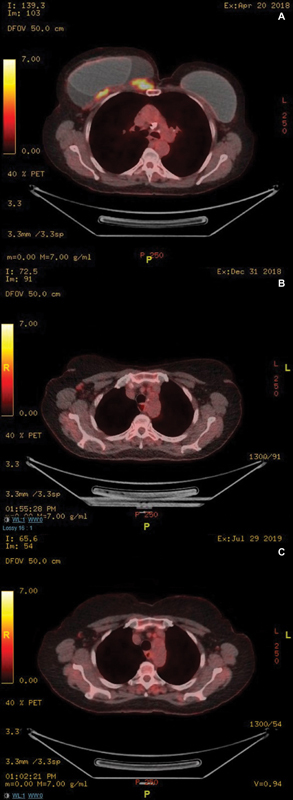
( **A** ) PET-CT at presentation demonstrating large peri-implant seroma and metabolically active parasternal mass infiltrating pectoralis muscle and closely attached to rib and sternum. ( **B** ) PET-CT after 4 cycles of BV. ( **C** ) PET-CT after 8 cycles of BV. BV, brentuximab vedotin.

A 65-year-old woman presented with progressive right breast swelling and palpable mass in the upper-inner quadrant ( Fig. 8 ). Nine years earlier, she underwent bilateral mastopexy augmentation with silicone macrotextured implants but denied previous breast problems. She had a history of chronic obstructive pulmonary disease, rosacea, and pulmonary tuberculosis in childhood. She underwent a hysterectomy and two cesarean sections. Her regular medications included formoterol and salbutamol inhalers and lymecycline. She was a long-term smoker and had a family history of cervical cancer, but no history of breast cancer or lymphoproliferative disease. Her physical examination revealed inverted T-incisions (Wise pattern) and a swollen right breast with a palpable 5 cm mass in the parasternal area, which was firm and fixed to the sternocostal junction and third rib. A breast ultrasound confirmed a large seroma, which was aspirated, and cytological features were suspicious, but inconclusive for BIA-ALCL. Subsequent MRI and CT revealed a 5 × 4 × 3 cm mass infiltrating the underlying pectoralis muscle. The diagnosis of BIA-ALCL was confirmed on core biopsy from the chest wall mass, which showed large, atypical cells with strong expression of CD30, some staining with epithelial membrane antigen and CD45, but negative for CD5, CD20, CD79a, and epithelial markers. Further immunochemistry tests were positive for CD4, but negative for ALK1, PAX5, CD2, CD7, and CD8. A PET-CT confirmed FDG-avid (SUV max 7.2) mass infiltrating beyond the implant capsule (stage IIA, T4N0M0). The anticipated extent of surgical procedure at this stage involved excision of the mass en bloc with capsulectomy and partial resection of ribs and sternum. In view of the significant morbidity and high risk of surgery, neoadjuvant therapy with BV was recommended to downstage the disease and ensure radical excision. The interval PET-CT after the first four cycles of BV showed a Deauville 1 metabolic response with residual mass, therefore further four cycles of BV were administered. Subsequent PET-CT confirmed a complete metabolic response and a significant reduction in the size of the mass. A surgical procedure was performed at this point, involving bilateral en bloc capsulectomy and implant removal (Allergan CUI 410 mL). Small peri-implant seroma was also aspirated intraoperatively. At surgery, there was no evidence of pectoralis muscle infiltration with a clear dissection plane around the capsule, however, multiple muscle biopsies were performed for histology. The surgical specimen, chest wall biopsies, and immunochemistry tests of the seroma did not reveal any residual disease. In view of the complete pathological response, no further immunotherapy or radiotherapy was recommended. The patient made uneventful postoperative recovery; however, she had myocardial infarction 2 months after surgery, which required coronary stenting and anticoagulation ( Fig. 9 ). The PET-CT performed 8 months after surgery confirmed complete metabolic remission as previously reported. 11 She is now 4 years postsurgery and remains well with no evidence of recurrence ( Fig. 10 ).

## Discussion

BIA-ALCL is an uncommon and emerging form of peripheral T-cell lymphoma diagnosed in patients with a history of textured breast implants. The etiology of this condition is still poorly understood, however current evidence indicates possible transformation and clonal expansion of deregulated immune cells as a response to chronic exposure to inflammatory cytokines in a genetically susceptible individual. 8 12 The majority of patients present at early stages of the disease with peri-implant effusion and breast swelling. In the systematic review by Leberfinger et al, 66% of patients were seen initially with seroma, 8% with a mass, 7% with mass and seroma, and 18% with other symptoms such as capsular contracture, axillary lymphadenopathy, and skin lesions. 13 One of our cases presented with skin rush, mass, and lymphadenopathy whereas the other with mass and seroma. Surgical resection remains the standard of care for patients with BIA-ALCL and includes explantation, total intact capsulectomy, and en bloc resection of all associated masses and involved lymph nodes. The technical aspects of this procedure were described by Tevis et al, 14 who recommended en bloc resection of breast implants with surrounding capsules, utilizing existing scars. Some patients may require skin excision in case of local involvement or close proximity of the mass, which may be incorporated in planned Wise-pattern mastopexy to reduce redundant skin after explantation but may occasionally require a separate incision. Tumescence of the posterior part of the capsule during total capsulectomy can be useful in the case of subpectoral implants, where the capsule is usually densely adherent to the periosteum and ribs, and inadvertent injury can lead to pneumothorax. 14 15 Every effort should be made to remove the entire capsule and avoid spillage of malignant effusion into the cavity. To mitigate this risk, an ultrasound-guided aspiration of the seroma just before surgery may be considered. The oncological radicality of surgery is essential for successful long-term outcomes as evidenced in several publications. 8 15 16 Clemens et al reported improved overall survival (OS; *p* < 0.001) and event-free survival (EFS) in patients treated with complete surgical excision compared with other therapeutic interventions. Only 5% of patients treated with complete excision had further events, which underlines the importance of achieving clear margins, regardless of adjuvant therapy regimens. However, the stage of BIA-ALCL at diagnosis had a significant effect on EFS. The rate of events was 2.6-fold higher for stage II and 2.7-fold higher for stage III disease compared with stage I disease in this case series. 17 Moreover, the size of the tumor and local invasion adversely affect the prognosis, as indicated by the event rate of 14.3% for stage T4 compared with 0% for stages T1/T2, even if complete resection was achieved. 8 17 In a recent systematic review including 178 BIA-ALCL cases, Naga et al found that presentation with mass as opposed to a seroma was significantly associated with recurrence and mortality (odds ratio of 19.4). 18 Management of locally advanced or surgically unresectable BIA-ALCL poses a significant challenge and requires a multidisciplinary approach. Early experience with the use of systemic chemotherapy and regimens including cyclophosphamide, doxorubicin, vincristine, and prednisolone, showed variable response. In a study of 87 BIA-ALCL cases published by Clemens et al, 17 over 50% of patients received chemotherapy and approximately one-third of these patients experienced progression of the disease or did not achieve a response. Eleven patients were treated with systemic chemotherapy and never underwent complete surgical excision, and six of these patients died as a result of the disease. The authors concluded that systemic chemotherapy alone was insufficient to control the disease. A retrospective review of 39 patients with locally advanced BIA-ALCL (stage IIB–IV) by Collins et al 19 showed much lower rates of definitive surgery (59%), higher mortality (20.8%), and lower rates of complete remission (71.8%) in this group compared with early-stage BIA-ALCL. Very rapid disease progression (hyperprogression) was observed in five patients (12.8%) who had only limited surgery, indicating a possible detrimental effect of incomplete BIA-ALCL excision. 18 Poor outcomes of locally advanced BIA-ALCL prompted interest in neoadjuvant systemic therapies to downstage the disease and enable complete surgical excision. Recently published NCCN guidelines 7 recommend adjuvant chemotherapy in advanced BIA-ALCL (stage II–IV) with either CHOP, CHOEP, or CHP + BV regimens. The efficacy of immunochemotherapy in the treatment of CD30+ peripheral T-cell lymphomas was also demonstrated in the ECHELON II trial, which exhibited statistically superior progression-free survival and OS compared with standard anthracycline-based chemotherapy. 19 20 Single-agent immunotherapy with BV as CD30 targeting antibody–drug conjugate is also an interesting therapeutic option, particularly for nonresponders to standard chemotherapy. 21 22 BV is an antibody–drug conjugate composed of an anti-CD30 chimeric antibody conjugated to monomethyl auristatin E, the microtubule-disrupting agent. The primary mechanism of action is targeted delivery of monomethyl auristatin E to CD30-expressing tumor cells. Additional proposed mechanisms that may contribute to the clinical activity of BV include antibody-dependent cellular phagocytosis and immunogenic cell death. However, the data on the treatment of BIA-ALCL in neoadjuvant settings are limited and based mainly on case reports ( Table 1 ). 11 16 23 24 25 Therapeutic decisions are extrapolated from experience with systemic and refractory ALCL, due to a lack of prospective studies and rarity of BIA-ALCL. Coombs et al reported on two cases of locally advanced BIA-ALCL (stage III) treated with neoadjuvant therapy (CHOEP and BV) with excellent clinical and pathological response. Both patients presented with large tumors and underwent extensive surgical procedures, including chest wall reconstructions, due to hypermetabolic activity on interval PET-CT and concerns about possible residual disease, however, final results confirmed a complete pathological response. 16 Stack and Levy 22 described the complete clinical and radiological response to monotherapy with BV in a case of unresectable ALCL. The patient received also palliative radiation therapy and remained in remission at 20 months after therapy. A combination of BV and vincristine was successfully used in neoadjuvant settings by Caputo et al 24 in the case of massive, fungating mass-like BIA-ALCL, infiltrating pectoralis muscle (stage IIA). The patient underwent radical excision followed by adjuvant BV and COMP chemotherapy and remained in complete remission for 12 months. Excellent response to neoadjuvant chemotherapy (CHOEP) in stage IV BIA-ALCL was also reported by Thibodeau et al. 23 Despite significant complications (pulmonary embolism and pancytopenia), the patient completed five cycles of CHOEP with complete resolution of hypermetabolic areas on PET-CT and no residual disease in the surgical specimen. All the above case reports are consistent with our experience with neoadjuvant therapy in two cases of advanced BIA-ALCL.

**Table 1:** Literature review on neoadjuvant therapy in locally advanced breast implant-associated anaplastic large cell lymphoma

Author	Year	Stage	Neoadjuvant therapy	Surgery	Pathological response	Adjuvant therapy	Outcome
Thibodeau et al 23	2019	IV	CHOEP 5 cycles	Bilateral explantation + en bloc capsulectomies. Wire-guided excision of left axillary and lateral breast masses	Complete	No	Complete remission at 8 months posttherapy
Allchin et al 11	2020	IIA	BV 8 cycles	Bilateral explantation + right en bloc capsulectomy + chest wall biopsies	Complete	No	Complete remission at 18 months postsurgery
Caputo et al 24	2021	IIA	BV + Vincristine	Excision of fungating, necrotic tumor en bloc with implant and pectoralis muscle. Dermal substitute for coverage and subsequent skin grafting	Partial/Complete excision + skin grafting	Yes: BV + COMP 5 cycles	Complete remission for 12 months/Died of cardiovascular disease
Coombs et al 16	2021	III	CHOEP 4 cycles	Initial open biopsy for diagnosis. Definitive procedure after neoadjuvant therapy—left anterior capsulectomy, partial breast excision, anterior chest wall resection (including third to fifth ribs) + reconstruction with Prolene mesh and methacrylate	Complete	No	Complete remission at 4 years postsurgery
		III	BV 8 cycles	Incisional biopsy of right chest wall mass. Definitive surgery after completion of neoadjuvant immunotherapy—chest wall mass resection (including third to fifth ribs) + reconstruction with pedicled latissimus dorsi flap + Prolene mesh and methacrylate. Postoperative hematoma and pleural effusion requiring drainage	Complete	No	Complete remission at 3 years postsurgery
Premji et al 25	2022	III	CHP + BV 6 cycles	Resection of right chest wall tumor (including third to fifth ribs), partial sternectomy, thymectomy, en bloc removal of the implant + reconstruction with mesh/bone cement and serratus advancement flap. Postoperative chest wall incision necrosis requiring debridement, washout, and tissue transfer	Partial/Complete excision + chest wall reconstruction	Yes: Radiotherapy Auto-SCT BV (12 cycles)	Complete remission at 1-year post auto-SCT

Abbreviations: Auto-SCT, autologous stem cells transplant; BV, brentuximab vedotin; CHOEP, cyclophosphamide, doxorubicin, vincristine, etoposide, prednisolone; CHP, cyclophosphamide, doxorubicin, prednisone; COMP, cyclophosphamide, liposomal doxorubicin, vincristine, prednisone, rituximab.

The limited sample size of this study and the paucity of literature regarding neoadjuvant therapeutic regimens underline the need for larger-scale studies to validate current findings. Although the results of the present case report as well as those reported in the literature are promising, surgical management remains the standard care of locally advanced BIA-ALCL and should always be performed in accordance with the most recent guidelines in the best interest of the patient. Similar to the patients reported in this study, suitable candidates for neoadjuvant therapeutic regimens include locally advanced cases of BIA-ALCL with surgically unresectable disease due to extension in the chest wall, patients with lymph node involvement, and those suitable to undergo chemoimmunotherapy or immunotherapy alone. The potential benefits of administering neoadjuvant therapeutic regimens may promote surgical de-escalation, in terms of a reduction in the extent of surgical intervention and change its timing. It is, however, important to underline that neoadjuvant therapies do not indicate avoidance surgery and must be performed in accordance with current guidelines. Therefore, tumor downstaging signifies a reduction in surgical extent as well as potential side effects associated with surgery.

To assess the long-term outcomes of immunotherapy in locally advanced BIA-ALCL, the patients should be closely monitored according to NCCN guidelines: clinical and radiological follow-up every 3 to 6 months for the first 2 years and subsequently annual review until 5 years posttherapy. The interval PET-CT provided important information about response and determined the duration of treatment and timing of surgical intervention. Complete surgical excision and complete pathological response were achieved in both patients after PET-CT confirmed the resolution of metabolically active disease. Both patients received BV, which proved to be very effective as single-agent or combined therapy. Several authors reported that BV is a potential alternative to cytotoxic chemotherapy and is characterized by excellent response rates and a favorable tolerability profile. 16 22 24

### Conclusion

The optimal management of patients with advanced BIA-ALCL and possible chest wall invasion remains unclear. Although surgery is an essential part of treatment, the extent and timing of surgical intervention should be carefully planned and warrant multidisciplinary discussion, especially when primary, radical resection is uncertain. We believe that in such cases, neoadjuvant therapy should be strongly considered. Monitoring of the response to neoadjuvant therapy is essential and may necessitate modification of the treatment plan.

Our experience supports the concept of neoadjuvant therapy in the management of locally advanced BIA-ALCL, which can offer downstaging of the disease, de-escalation of surgery, and reduce the risk of significant complications. Based on emerging evidence, targeted immunotherapy with BV as monotherapy or in combination with chemotherapy, seems to be a preferred induction therapy in such cases, offering good response rates and an acceptable tolerance profile. Further genomic research and clinical data from larger, controlled studies may provide further evidence on patient selection and optimal management of locally advanced BIA-ALCL.

## References

[1] Swerdlow S H Campo E Pileri S A The 2016 revision of the World Health Organization classification of lymphoid neoplasms Blood 2016 127 20 2375 2390 26980727 10.1182/blood-2016-01-643569 PMC4874220

[2] Nava M B Adams W P Jr Botti G MBN 2016 Aesthetic Breast Meeting BIA-ALCL Consensus Conference Report Plast Reconstr Surg 2018 141 01 40 48 29280860 10.1097/PRS.0000000000003933

[3] Keech J A Jr Creech B J Anaplastic T-cell lymphoma in proximity to a saline-filled breast implant Plast Reconstr Surg 1997 100 02 554 555 9252643 10.1097/00006534-199708000-00065

[4] American Society of Plastic Surgeons.Breast Implant-Associated Anaplastic Large Cell Lymphoma (BIA-ALCL) 2024. Accessed 21 April 21, 2024 at:https://www.plasticsurgery.org/patient-safety/breast-implant-safety/bia-alcl-summary

[5] Zhang X R Chien P N Nam S Y Heo C Y anaplastic large cell lymphoma: molecular pathogenesis and treatment Cancers (Basel) 2022 14 07 1650 35406421 10.3390/cancers14071650 PMC8997054

[6] Turton P El-Sharkawi D Lyburn I UK Guidelines on the Diagnosis and Treatment of Breast Implant-Associated Anaplastic Large Cell Lymphoma (BIA-ALCL) on behalf of the Medicines and Healthcare products Regulatory Agency (MHRA) Plastic, Reconstructive and Aesthetic Surgery Expert Advisory Group (PRASEAG) J Plast Reconstr Aesthet Surg 2021 74 01 13 29 33483089 10.1016/j.bjps.2020.10.064

[7] Horwitz S M Ansell S Ai W Z T-cell lymphomas, Version 2.2022, NCCN Clinical Practice Guidelines in Oncology J Natl Compr Canc Netw 2022 20 03 285 308 35276674 10.6004/jnccn.2022.0015

[8] U.S. Food and Drug Administration ǀ Center for Devices and Radiological Health.CDRH's Continued Commitment to Breast Implant SafetyFebruary 28,2024. Accessed April 21, 2024 at:https://www.fda.gov/medical-devices/medical-devices-news-and-events/cdrh-statement-cdrhs-continued-commitment-breast-implant-safety#:~:text=Given%20that%20the%20occurrence%20of,informed%20decision%20about%20their%20health

[9] Clemens M W Jacobsen E D Horwitz S M 2019 NCCN Consensus Guidelines on the Diagnosis and Treatment of Breast Implant-Associated Anaplastic Large Cell Lymphoma (BIA-ALCL) Aesthet Surg J 2019 39 01 S3 S13 30715173 10.1093/asj/sjy331

[10] Italian Ministry of Health.Linee di indirizzo sul percorso diagnostico terapeutico assistenziale per il linfoma anaplastico a grandi cellule in pazienti con impianti protesici mammari (BIA-ALCL)[Guidelines on the diagnostic and therapeutic management of patients with breast implant-associated anaplastic large cell lymphoma (BIA-ALCL)].2022. Accessed October 15, 2023 at:https://sirm.org/wp-content/uploads/2022/11/Linee-indirizzo-_LINFOMA-ANAPLASTICO-A-GRANDI-CELLULE-IN-PAZIENTI-CON-IMPIANTI-PROTESICI-MAMMARI-BIA-ALCL.pdf

[11] Allchin R L Wickenden K Pilgrim S Wilson-Morkeh I Miall F M The successful use of neo adjuvant brentuximab vedotin in the treatment of BIA-ALCL HemaSphere 2020 4 06 e501 33283170 10.1097/HS9.0000000000000501 PMC7710186

[12] DeCoster R C Clemens M W Di Napoli A Cellular and molecular mechanisms of breast implant-associated anaplastic large cell lymphoma Plast Reconstr Surg 2021 147 01 30e 41e 10.1097/PRS.0000000000007423 33370049

[13] Leberfinger A N Behar B J Williams N C Breast implant-associated anaplastic large cell lymphoma: a systematic review JAMA Surg 2017 152 12 1161 1168 29049466 10.1001/jamasurg.2017.4026

[14] Tevis S E Hunt K K Clemens M W Stepwise en bloc resection of breast implant-associated anaplastic large cell lymphoma with oncologic considerations Aesthet Surg J Open Forum 2019 1 01 ojz005 33791601 10.1093/asjof/ojz005 PMC7984833

[15] Clemens M W Brody G S Mahabir R C Miranda R N How to diagnose and treat breast implant-associated anaplastic large cell lymphoma Plast Reconstr Surg 2018 141 04 586e 599e 10.1097/PRS.0000000000004262 29595739

[16] Coombs D M Aliotta R Jagadeesh D Raymond D Isakov R Breast implant-associated anaplastic large cell lymphoma with invasive chest wall masses Ann Plast Surg 2021 87 04 409 414 34176904 10.1097/SAP.0000000000002910

[17] Clemens M W Medeiros L J Butler C E Complete surgical excision is essential for the management of patients with breast implant-associated anaplastic large-cell lymphoma J Clin Oncol 2016 34 02 160 168Erratum in: J Clin Oncol. 2016 Mar 10;34(8):888. DiNapoli, Arianna [corrected to Di Napoli, Arianna]. PMID: 26628470; PMCID: PMC4872006 26628470 10.1200/JCO.2015.63.3412 PMC4872006

[18] Naga H I Mellia J A Basta M N Breast implant-associated anaplastic large-cell lymphoma: updated systematic review and analysis of treatment strategies Plast Reconstr Surg 2022 150 04 762 769 35862104 10.1097/PRS.0000000000009538 PMC9551598

[19] Collins M S Miranda R N Medeiros L J Characteristics and treatment of advanced breast implant-associated anaplastic large cell lymphoma Plast Reconstr Surg 2019 143(3S A Review of Breast Implant-Associated Anaplastic Large Cell Lymphoma):41S 50S 10.1097/PRS.0000000000005568 30817555

[20] Horwitz S O'Connor O A Pro B The ECHELON-2 Trial: 5-year results of a randomized, phase III study of brentuximab vedotin with chemotherapy for CD30-positive peripheral T-cell lymphoma Ann Oncol 2022 33 03 288 298 34921960 10.1016/j.annonc.2021.12.002 PMC9447792

[21] Alderuccio J P Desai A Yepes M M Chapman J R Vega F Lossos I S Frontline brentuximab vedotin in breast implant-associated anaplastic large-cell lymphoma Clin Case Rep 2018 6 04 634 637 29636930 10.1002/ccr3.1382 PMC5889253

[22] Stack A Levy I Brentuximab vedotin as monotherapy for unresectable breast implant-associated anaplastic large cell lymphoma Clin Case Rep 2019 7 05 1003 1006 31110735 10.1002/ccr3.2142 PMC6510013

[23] Thibodeau R Fan K L Wehner P B Stage IV breast implant-associated anaplastic large-cell lymphoma with complete pathologic response to neoadjuvant chemotherapy Plast Reconstr Surg Glob Open 2019 7 09 e2446 31942403 10.1097/GOX.0000000000002446 PMC6908402

[24] Caputo G G Alban A D'Alì L Mariuzzi L Galvano F Parodi P C Locally advanced breast implant-associated anaplastic large-cell lymphoma: a combined medical-surgical approach Eur Rev Med Pharmacol Sci 2021 25 09 3483 3488 34002822 10.26355/eurrev_202105_25830

[25] Premji S Barbieri A Roth C Rohren E M Rivero G Teegavarapu S P An unusual case of breast implant-associated anaplastic large cell lymphoma Case Rep Hematol 2022 2022 4.700787E6 10.1155/2022/4700787 PMC9203203 35721802

